# Supplementary material for: A preclinical secondary pharmacology resource illuminates target-adverse drug reaction associations of marketed drugs
Source: Nat Commun. 2023 Jul 19;14:4323. doi: 10.1038/s41467-023-40064-9 (PMC10356841; doi:10.1038/s41467-023-40064-9)
Supplement: Supplementary file 1 — Supplementary Information [file 41467_2023_40064_MOESM1_ESM.pdf]

## Supplementary Materials

# A preclinical secondary pharmacology resource illuminates target-adverse drug reaction associations of marketed drugs

**Authors:**
Jeffrey J Sutherland[1], Dimitar Yonchev[2], Alexander Fekete[1], Laszlo Urban[1,*]

**Affiliations:**
[1]Novartis Institutes for Biomedical Research, Cambridge, MA, United States
[2]Novartis Institutes for Biomedical Research, Basel, Switzerland
* Corresponding author laszlo.urban@novartis.com

## Supplementary Notes

**Investigation of FAERS likelihood ratio test (LRT) threshold**

Drug vs. ADR risk from FAERS are annotated with a LRT statistic in DrugCentral. Increasing the LRT threshold for distinguishing drugs annotated as positive (above the LRT) for a given ADR may focus on the smaller set of drugs with higher incidence of the ADR, at the expense of reducing the count of drugs annotated as positive and hence power to detect a significant relationship. The selection of a threshold is arbitrary, values such as 2, 5 or 10 may be selected. Throughout this work, LRT threshold of 5 was used.

To investigate the impact of using a different threshold, the statistical significance of the literature-reported target-ADR relationships from Supplementary Data 6 was evaluated separately using SIDER, FAERS LRT threshold of 2 and 5. Criteria for significance were the same as Fig. 3, namely KW p-value $\leq 0.001$ and ROC AUC $\geq 0.6$. Because we required at least 10 ADR positives with assay results when evaluating each target-ADR relationship, and SIDER vs. FAERS or LRT 2 vs. 5 affects this count, 539 target-ADR pairs with assessed significance on the 3 methods were retained for analysis (Supplementary Data 14). The 3 methods were compared via 2 x 2 contingency tables and $\chi^2$ tests (Supplementary Table 2). Of 114 target-ADR pairs significant at LRT threshold of 5, 86 were also significant at LRT threshold of 2. While all 3 pairs of approaches are highly concordant by the $\chi^2$ statistic, results are more similar when comparing the FAERS LRT thresholds than FAERS vs. SIDER. As such, different LRT thresholds result in broadly similar conclusions.

**Investigation of assay activity thresholds for defining assay positives vs. negatives**

Observing a statistically significant relationship for a given target and ADR does not provide guidance on its use for assessing compounds. For example, the relationship between hERG (KCNH2) and "Prolongation of QT interval" (MedDRA 10014387) has KW-p-value 1.6e-13 and ROC AUC 0.68 (assessed using SIDER; Supplementary Data 6). Our preferred approach is to report the odds of observing the ADR in clinical use given the measured level of activity; there is no benefit in declaring compounds as being active or inactive in the hERG assay by thresholding on the free margin or $AC_{50}$. When thresholding is applied, there is a trade-off between sensitivity (identifying all the QT prolonging drugs) and specificity (falsely labelling a safe drug as QT prolonging). The threshold may change during drug discovery, with a preference for avoiding false positives in later stages. As such, the partial ROC AUC assessed at high specificity may be preferred over the full AUC. To investigate the impact of this threshold, we calculated the partial ROC AUC over the 90-100% specificity interval, and compared to the full (standard) ROC AUC and KW p-value over the full dataset of assay vs ADR pairs (Supplementary Fig. 6; dataset produced by the calc_AE_vs_assay_score.ipynb notebook). The high correlation observed indicates that results using this partial AUC would be broadly comparable to those using the standard AUC. We favor the standard AUC because of its familiarity and simple interpretation: the ROC AUC conveys the probability that a randomly selected drug positive for an ADR is ranked above a randomly selected negative drug. A ROC AUC of 0.5 indicates a random result. Partial AUCs are smaller because they measure a fraction of the full specificity interval, and there is no single standard cutoff like 0.5 that corresponds to random accuracy.

**Investigation of stability of variable selection in multivariate modelling of adverse drug reactions**

Lasso-penalized logistic regression modelling was used to select non-redundant variables (assay and activity measure) explaining outcomes for each source (SIDER or FAERS) and MedDRA code: 115 ADR models from FAERS and 259 from SIDER. For each model, the optimal value of the shrinkage L1 penalty (parameter "C" in scikit-learn LogisticRegression) was selected by performing 50 trials of leave 20% out cross validation and identifying the most penalized model (smallest "C") within 1 standard error of the maximal ROC AUC. A single final model was subsequently created at the optimal parameter using the full dataset, and variables having coefficient ≤ -0.08 in this single model were labelled as non-redundant in explaining the ADR (Supplementary Data 7 column "parameters in sparse model"). It should be noted that Supplementary Data 7 summarizes inclusion of assays using any of the three activity measures: free margin, total margin or unadjusted AC50. Variables as used in the model are a combination of assay and activity measure, e.g. KCNH2 AC50 and KCHN2 free margin are separate variables, only one of which might be selected as non-redundant owing to their correlation.
To investigate whether the variables selected as non-redundant would change with variation in the dataset, we compared the coefficient in the single final model to the frequency of that variable's inclusion across the 50 repeats (i.e. selecting variables on the training sets only, inside the cross validation loop). For FAERS, 72% of non-redundant predictors were reselected in 40 or more of the 50 repeats, and 81% for SIDER; 2-4% were re-selected fewer than 25 repeats (Supplementary Table 3). Further, of 221 variables re-selected in fewer than 40 repeats, 63

(29%) involved assays that were re-selected using a different activity measure, e.g. retaining the use of KCNH2 assay, but using free margin instead of total margin (Supplementary Data 15). This indicates that the assays selected as non-redundant predictors of ADR risk, as tabulated in Supplementary Data 6 and elsewhere, are not sensitive to variation in the derivation data.

# Supplementary Tables

**Supplementary Table 1. Investigation of clinical adverse drug reactions attributed to activity in safety pharmacology targets**

| Target | Mode | % sig. (total)[a] | Significant ADRs[b] |
|---|---|---|---|
| ACHE | inhibition | 6 (33) | salivation ↑, tingling/weakness in limbs |
| ADRA1A | activation | 67 (6) | BP ↑, cardiac arrhythmia, pupil diameter ↑, smooth muscle contraction |
| ADRA1A | inhibition | 78 (9) | BP ↓, dizziness, heart rate ↑, impact on various aspects of sexual function, orthostatic hypotension, retrograde ejaculation, smooth muscle tone ↓ |
| ADRA2A | activation | 44 (9) | heart rate ↓, pupil diameter ↑, sedation |
| ADRA2A | inhibition | 20 (5) | heart rate ↑ |
| ADRA2B | activation | 43 (7) | heart rate ↑, sedation, skeletal muscle tremor |
| ADRB1 | activation | 38 (8) | bronchospasm, heart failure, ventricular fibrillation |
| ADRB1 | inhibition | 50 (2) | heart rate ↓ |
| ADRB2 | activation | 67 (9) | bronchospasm, cardiac arrest ↑, heart failure, heart rate ↑, QTC interval ↑, skeletal muscle tremor |
| ADRB2 | inhibition | 50 (2) | BP ↓ |
| AGTR1 | inhibition | 14 (7) | respiratory distress syndrome |
| AR | activation | 43 (7) | androgenicity in females, prostate carcinoma ↑ |
| AR | inhibition | 32 (19) | breast carcinoma ↑, insulin resistance, mastodynia, sexual dysfunction, spermatogenesis ↓ |
| CACNA1C | activation | 33 (3) | locomotor activity ↓ |
| CHRM1 | activation | 38 (29) | blurred vision, centrilobular liver congestion, exhaustion, heart rate ↑, irritability, locomotor activity ↓, ptosis, pupil diameter ↓, salivation ↑, tachycardia |
| CHRM1 | inhibition | 80 (5) | blurred vision, cognitive function ↓, heart rate ↑, locomotor activity ↑ |
| CHRM2 | activation | 45 (22) | blurred vision, cardiac action potential duration decrease, exhaustion, heart rate ↓, heart rate ↑, PR interval ↓, pupil diameter ↓, salivation ↑ |
| CHRM2 | inhibition | 80 (5) | cardiac conduction ↓, heart rate ↑, tachycardia, tremors |
| CHRM3 | activation | 20 (25) | blurred vision, bronchoconstriction, bronchospasm, heart rate ↑, pupil diameter ↓, urinary contraction |
| CHRM3 | inhibition | 89 (9) | blurred vision, constipation, dry mouth, GI motility ↓, interferes with ocular accommodation, intestinal transit ↓, pupil diameter ↑, salivation ↓↑ |
| CHRM4 | activation | 100 (1) | pupil diameter ↓ |
| CNR1 | activation | 6 (17) | drug abuse/dependence↓ |
| DRD1 | activation | 50 (16) | arousal ↑, drug abuse/dependence, dyskinesia, hypotension, locomotor activity ↓, locomotor activity ↑, psychosis |

| | | | |
|---|---|---|---|
| DRD1 | inhibition | 80 (10) | anxiety, coordination disorders, dyskinesia, locomotor activity ↓, parkinsonian symptoms (tremors), parkinsonism, suicidal intent |
| DRD2 | activation | 56 (16) | body temperature ↓, drowsiness, drug abuse/dependence, fainting, GI transit ↓, hallucinations, locomotor activity ↓, locomotor activity ↑, stereotypy |
| DRD2 | inhibition | 50 (8) | drowsiness, GI motility ↑, locomotor activity ↓, orthostatic hypotension |
| GABRA1 | activation | 53 (15) | anterograde amnesia, ataxia, dizziness, drug abuse/dependence, locomotor activity ↓, memory ↓, sedation, sleep ↑ |
| GABRA1 | inhibition | 50 (2) | convulsions |
| HRH1 | activation | 40 (15) | BP ↓, drinking ↑, facial swelling, flushing, sweating, tongue swelling |
| HRH1 | inhibition | 100 (11) | BP ↓, body weight ↑, cardiac arrhythmia, convulsions ↑, GI transit ↓, heart rate ↑, locomotor activity ↑, QTc interval ↑, sedation, sleep ↑ |
| HRH2 | activation | 33 (6) | drinking ↑, heart rate ↑ |
| HRH3 | inhibition | 25 (4) | sedation |
| HTR1A | activation | 71 (14) | adrenocorticotropic hormone ↑, body temperature ↓, growth hormone secretion ↑, locomotor activity ↓, locomotor activity ↑, pupil diameter ↓, pupil diameter ↑, reduced rapid eye movement sleep, sleep ↑, stereotypy |
| HTR1A | inhibition | 50 (6) | dizziness, locomotor activity ↑, anxiogenic |
| HTR2A | activation | 71 (17) | agitation, drug abuse/dependence, hallucinations, heart rate ↑, hyperreflexia, myoclonus, psychosis, pupil diameter ↑, schizophrenia, serotonin syndrome, smooth muscle contraction, stereotypy |
| HTR2A | inhibition | 33 (3) | sleep ↑ |
| HTR2B | activation | 33 (3) | cardiac valvulopathy |
| HTR2B | inhibition | 100 (1) | GI transit ↓ |
| HTR2C | activation | 71 (7) | abnormal mouth movements, anxiety ↑, convulsions ↑, locomotor activity ↓, penile erection |
| HTR2C | inhibition | 100 (1) | drug abuse/dependence |
| HTR3A | inhibition | 33 (6) | constipation, GI transit ↓ |
| KCNH2 | inhibition | 100 (1) | prolongation of QT interval of ECG |
| NR3C1 | activation | 67 (12) | blood glucose ↑, body weight ↑, glaucoma, hyperglycemia, insulin resistance, muscle mass ↓, osteoporosis, wound repair ↓ |
| OPRD1 | inhibition | 33 (3) | pain ↑ |
| OPRK1 | activation | 85 (20) | anxiety ↑, confusion, dizziness, drinking ↑, drug abuse/dependence, dysphoria, eating ↑, GI motility ↓, GI transit ↓, hallucinations, heart rate ↓, heart rate ↑, locomotor activity ↓, sedation, tachycardia |
| OPRK1 | inhibition | 100 (3) | convulsions ↑ |
| OPRM1 | activation | 40 (20) | drug abuse/dependence, GI motility ↓, GI transit ↓, pupil diameter ↓, pupil diameter ↑, respiratory depression, sedation |
| PTGS1 | inhibition | 75 (4) | dyspepsia, gastric bleeding, renal dysfunction |

| | | | |
|---|---|---|---|
| PTGS2 | inhibition | 33 (6) | urinary sodium excretion ↓ |
| SCN5A | activation | 60 (5) | cardiac arrhythmia, heart rate ↑, locomotor activity ↓ |
| SCN5A | inhibition | 55 (11) | cardiac arrhythmia, GI transit ↓, heart rate ↓, heart rate ↑ |
| SLC6A2 | inhibition | 47 (15) | constipation, drug abuse/dependence, locomotor activity ↓, locomotor activity ↑, pupillary reflex ↓, QTC interval ↑, urinary hesitancy |
| SLC6A3 | activation | 50 (2) | coordination ↓ |
| SLC6A3 | inhibition | 60 (10) | dyskinesia, dystonia, locomotor activity ↑, parkinsonism, psychostimulation, stereotypy |
| SLC6A4 | inhibition | 59 (17) | anxiety ↑, diarrhea/constipation, dizziness, GI motility ↑, locomotor activity ↓, locomotor activity ↑, sexual dysfunction, sleep ↓, tremor, upper GI transit ↓ |

[a] percent of associations having p-KW ≤ 0.001 (number of associations tested); [b] BP: blood pressure, GI: gastrointestinal; ↑: increased; ↓: decreased

**Supplementary Table 2. Contingency tables and $\chi^2$ tests comparing FAERS likelihood ratio test (LRT) thresholds of 2, 5 vs. SIDER on 539 literature-reported target-ADR pairs**

| **FAERS LRT5 vs LRT2: $\chi^2$ = 262, p = 5e-59** | | | |
|---|---|---|---|
| | | | |
| | | **FAERS LRT5 significant** | |
| | | No | Yes |
| **FAERS LRT2 significant** | No | 399 | 28 |
| | Yes | 26 | 86 |
| | | | |
| | | | |
| **FAERS LRT5 vs SIDER: $\chi^2$ = 143, p = 7e-33** | | | |
| | | | |
| | | **FAERS LRT5 significant** | |
| | | No | Yes |
| **SIDER significant** | No | 345 | 26 |
| | Yes | 80 | 88 |
| | | | |
| **FAERS LRT2 vs SIDER: $\chi^2$ = 190, p = 8e-43** | | | |
| | | | |
| | | **FAERS LRT2 significant** | |
| | | No | Yes |
| **SIDER significant** | No | 354 | 17 |
| | Yes | 73 | 95 |

**Supplementary Table 3. Frequency at which non-redundant ADR predictors are re-selected across 50 repeated train vs test splits**

| | **Percent (count) of variables[a]** | |
|---|---|---|
| **Variable frequency across 50 models** | **FAERS** | **SIDER** |
| 1-9 | 0% (1) | 0% (0) |
| 10-24 | 4% (13) | 2% (13) |
| 25-39 | 24% (80) | 17% (114) |
| 40-50 | 72% (245) | 81% (552) |

[a] frequency at which 339 variables selected as non-redundant predictors of FAERS ADRs and 679 variables as predictors of SIDER ADRs are re-selected across 50 random train/test splits

## Supplementary Figures

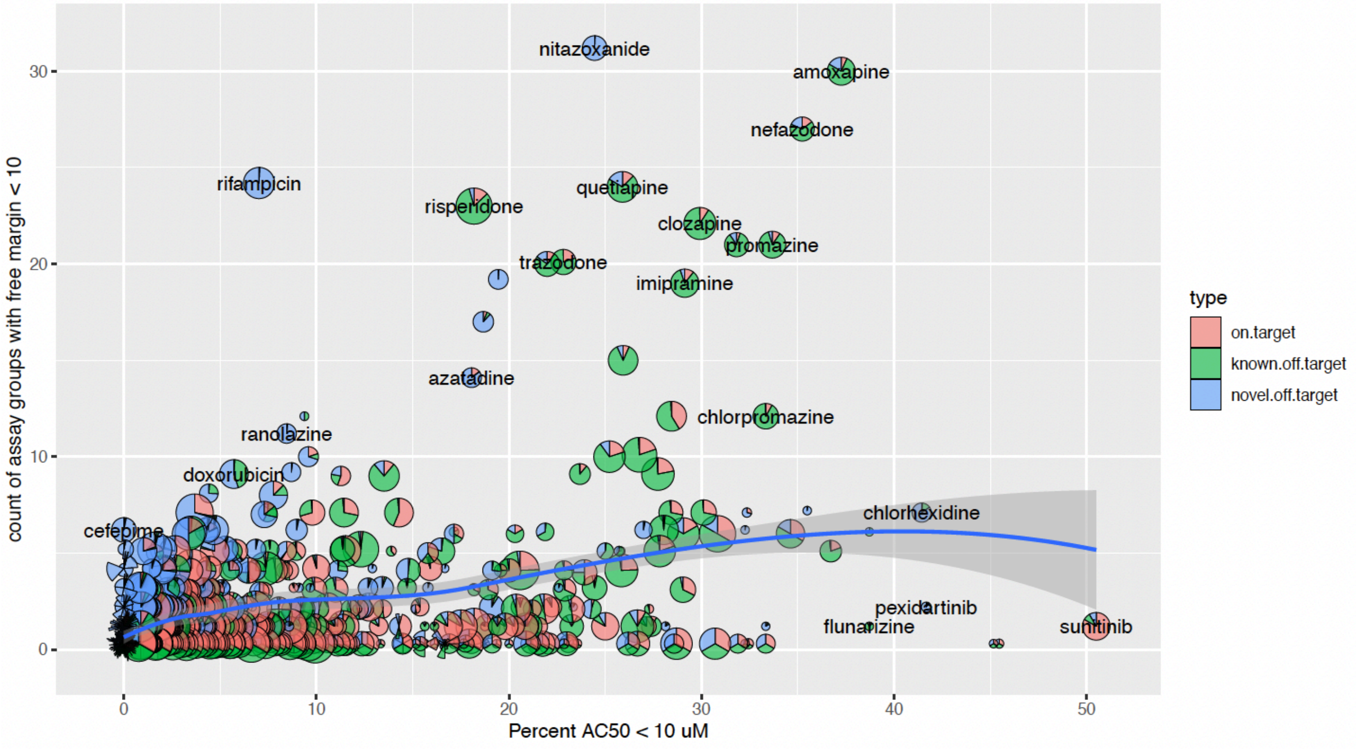


Supplementary Fig. 1. **Relationship between promiscuity derived from $AC_{50}$ and count of physiologically relevant activities.** Promiscuity (x-axis) is calculated as the percentage of AC50 results < 10 µM; only drugs with 30 or more assay results were included; count of physiologically relevant activities (y-axis) denotes assay results with free margin ≤ 10. Pie size is proportional to the count of assay results; pie distribution shows the proportion of physiological activities that are on-target (salmon), known off-targets (green) and unpublished off-target (blue). LOESS smoothed trend (moving average, blue) and 95% confidence intervals (gray). Compounds discussed in the text include nefazodone (31/88 assays with $AC_{50}$ results ≤ 10 µM, or 35%) with 27 physiological activities (4 on-target, 18 known off-target and 5 unpublished off-target activities: ADORA3, ADRB3, GHSR, MC3R, MC4R); cefepime has 6 unpublished off-target activities (ESR1, HRH3, NR1I2, PGR, PPARG, PTGS2).

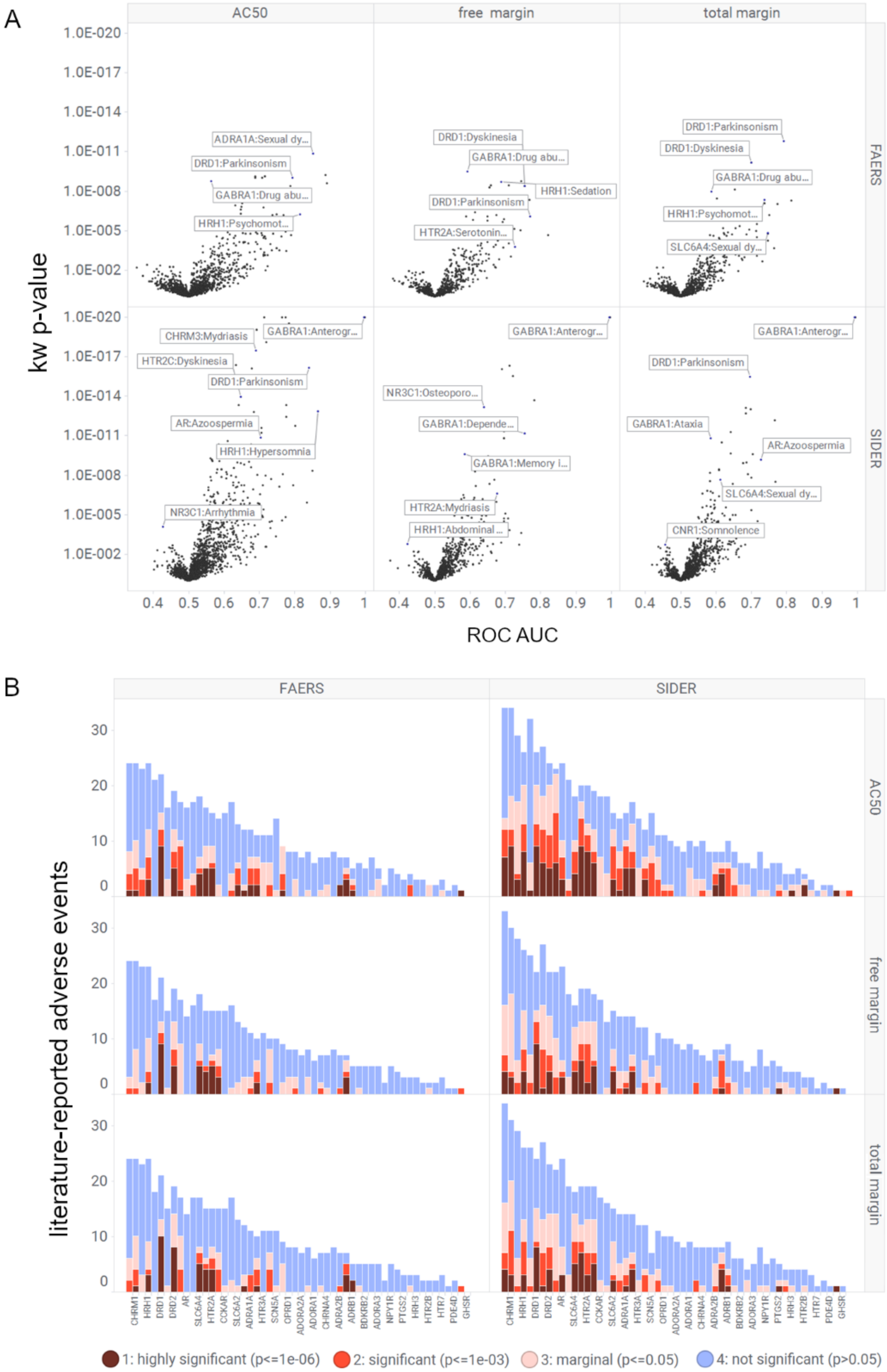


(Supplementary Fig. 2 legend on next page)

Supplementary Fig. 2. **Statistical significance of literature-reported target vs. ADR associations by activity measure and source.** A) Comparison of KW p-value vs. ROC AUC by activity measure (AC50, free margin or total margin) vs. source (SIDER, FAERS) for literature associations. B) Total number of adverse drug reactions reported across targets, distinguished by level of statistical significance observed.

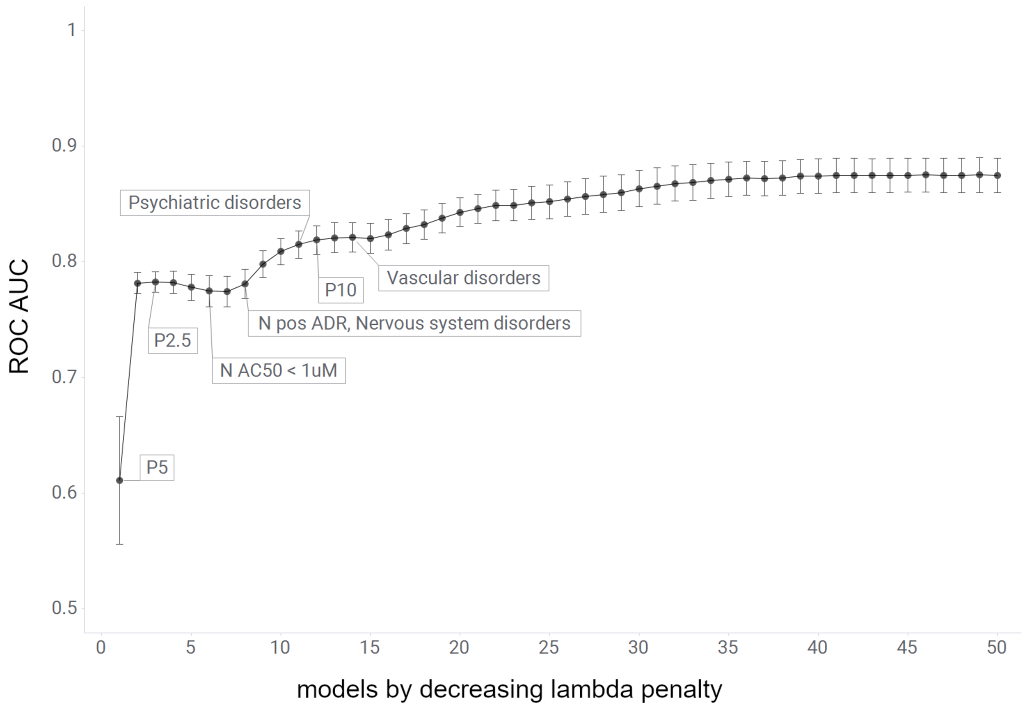


Supplementary Fig. 3**. Identification of attributes associated with significance of literature-reported target vs. ADR pairs.** Literature target-ADR pairs were labelled as significant ($p < 0.001$ and ROC AUC $\geq 0.6$) or non-significant (all others) based on the KW-test and ROC AUC analysis. Penalized (lasso) logistic regression was used to classify outcomes, with models consisting of 5 variables having cross-validated ROC AUC ~ 0.8. Variables selected for inclusion in the smaller models are labelled. Error bars are SEM on 50 repeated train/test splits; P2.5 = 2.5$^{th}$ percentile; P5 = 5$^{th}$ percentile; P10 = 10$^{th}$ percentile; Npos ADR = number of positive drugs for the ADR

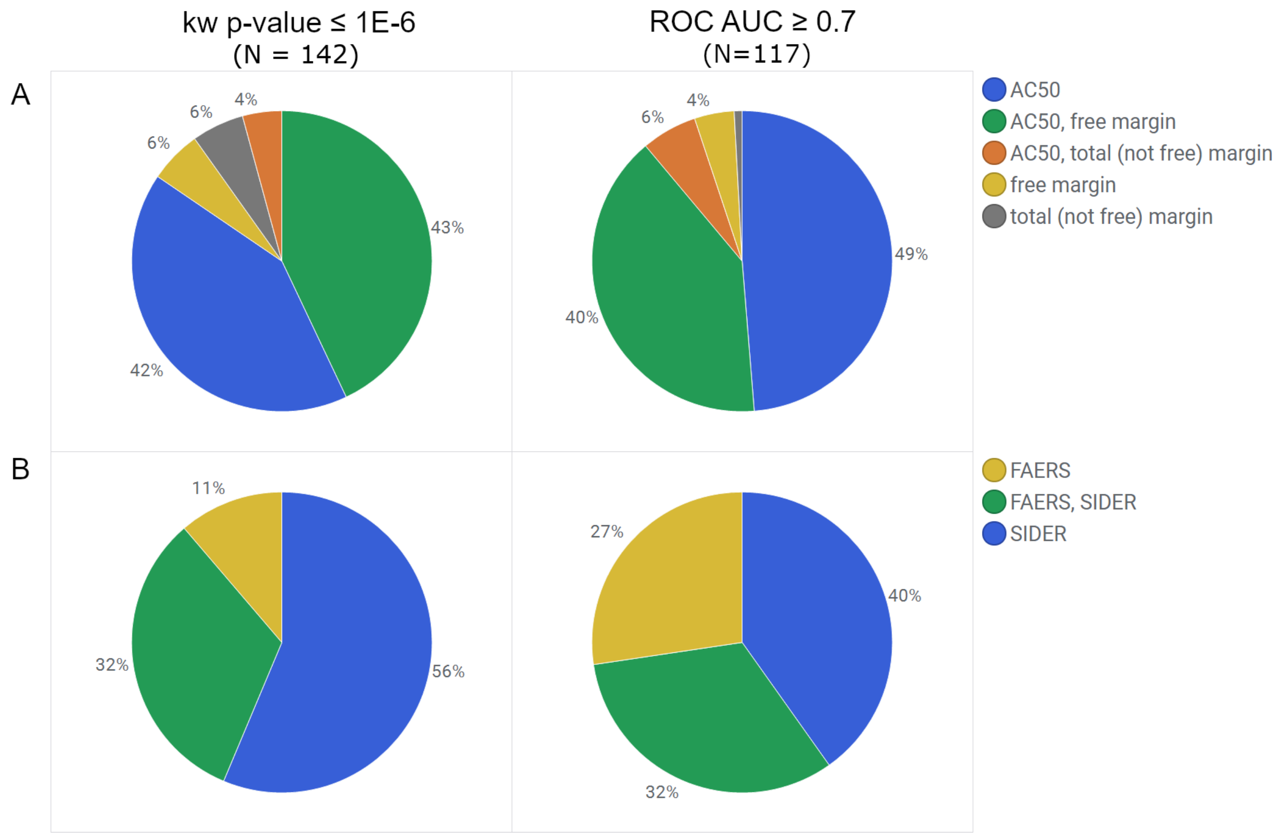


Supplementary Fig. 4. **Distribution of significant literature-reported target-ADR pairs.** A) Distribution by activity measure: pairs significant for AC50 only (blue), AC50 and free margin (green – total margin not considered), AC50 and total margin (orange – i.e. not significant on free margin), free margin only (yellow - i.e. not significant on AC50, total margin not considered) and total margin only (gray). To simplify the number of categories, association on total margin was only considered when free margin was not significant. B) Distribution by ADR source: pairs significant in FAERS only (yellow), FAERS and SIDER (green), SIDER only (blue). Significance was assessed separately on KW p-value (left panel) vs. ROC AUC (right panel).

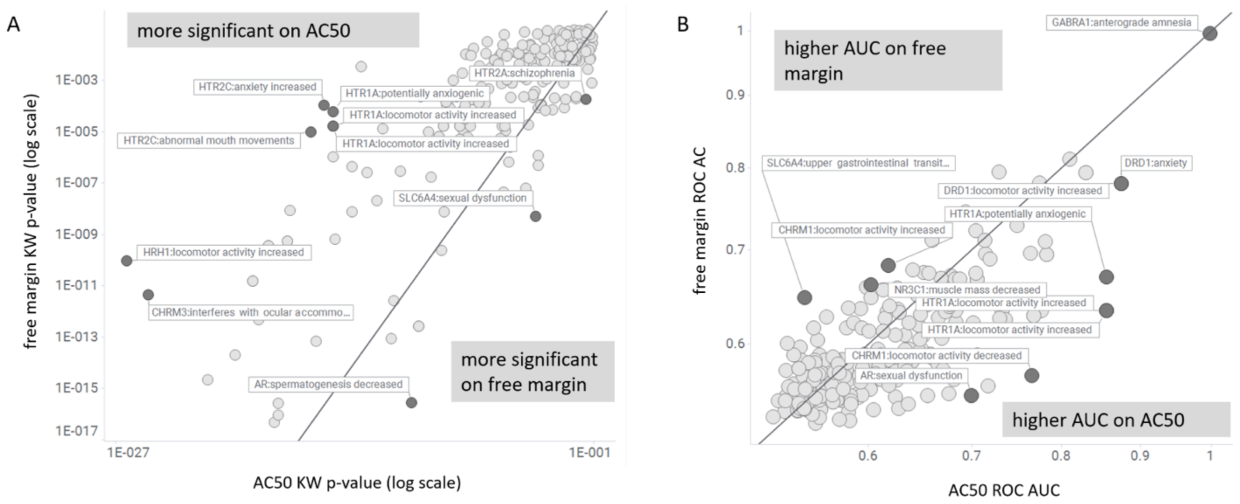


Supplementary Fig. 5. **Comparison of literature-reported target-ADR pairs assessed on free margin vs. AC50**. A) comparison using KW p-value and B) ROC AUC. Selected target-ADR pairs are labelled as gene symbol: MedDRA name

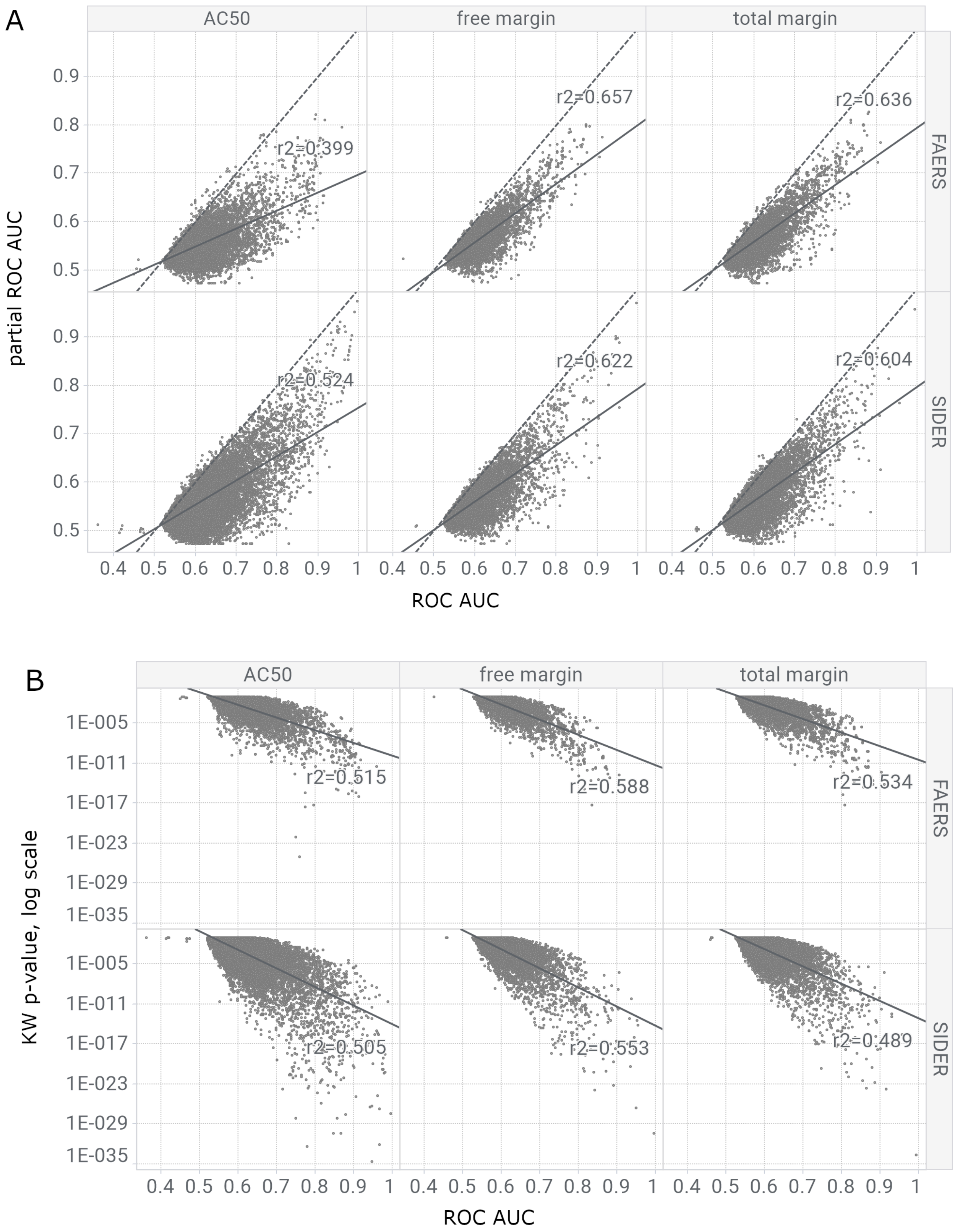


(Supplementary Fig. 6, continued next page)

C

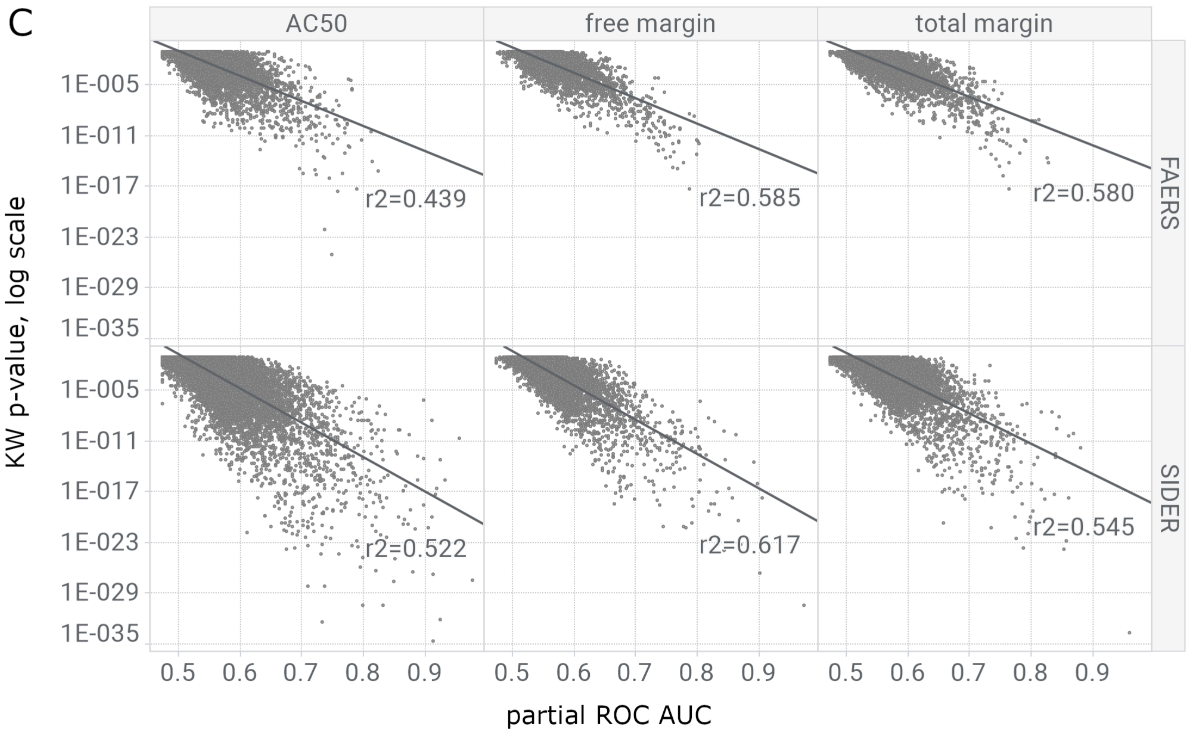


Supplementary Fig. 6. **Comparison of partial ROC AUC vs. standard ROC AUC and KW p-value.** Each point represents an assay-ADR pair, obtained via the systematic evaluation of all possible assay vs. ADR pairs using 2 sources (SIDER and FAERS) and 3 measures of activity (free margin, total margin, unadjusted $AC_{50}$) (Jupyter notebook calc_AE_vs_assay_score.ipynb with the default cutoff_dict settings). A) full ROC AUC vs. partial ROC AUC, B) full ROC AUC vs. KW p-value, C) partial ROC AUC vs. KW p-value. Solid line shows linear regression fit, dashed line (panel A) shows equality.
