# Supplementary material for: A preclinical secondary pharmacology resource illuminates target-adverse drug reaction associations of marketed drugs
Source: Nat Commun. 2023 Jul 19;14:4323. doi: 10.1038/s41467-023-40064-9 (PMC10356841; doi:10.1038/s41467-023-40064-9)
Supplement: Supplementary file 3 — Description of Additional Supplementary Files [file 41467_2023_40064_MOESM3_ESM.pdf]

Description of Additional Supplementary Files

Supplementary Data 1. Safety Pharmacology Database assay results

Supplementary Data 2. Unique drug substances tested in safety pharmacology assays

Supplementary Data 3. Safety pharmacology assays used for characterizing marketed drugs

Supplementary Data 4. Safety margin characteristics of on-target drug interactions

Supplementary Data 5. Comparison of on-target indications vs. top-10 indications associated with the novel off-target activity

Supplementary Data 6. Significance analysis of literature reported target vs. adverse event relationships

Supplementary Data 7. Assay vs. AE pairs from univariate analysis and annotation with multivariate model coefficients

Supplementary Data 8. Details of NSPD activities not reported in literature sources potentially associated with drugs' known clinical adverse events

Supplementary Data 9. Summary of NSPD activities not reported in literature sources potentially associated with drugs' known clinical adverse events

Supplementary Data 10. Dataset of matched SPD AC50 vs. individual ChEMBL AC50 results used to identify assay attributes contributing to activity differences

Supplementary Data 11. Mapping of chemical identifiers from SIDER / STITCH to DrugCentral

Supplementary Data 12. Literature-reported target vs. AE associations from 3 reviews and annotation with MedDRA terminology

Supplementary Data 13. Dataset of most significant AC50 result for each literature-reported target vs. ADR relationship

Supplementary Data 14. Significance analysis of literature reported target vs. adverse event relationships in SIDER vs FAERS at LRT thresholds of 2 and 5 (target-ADR pairs from Table S5)

Supplementary Data 15. Tabulation of non-redundant variable frequency in 50 repeated random sample models
